# Comparative evaluation of different molecular methods for DNA extraction from individual *Teladorsagia circumcincta* nematodes

**DOI:** 10.1186/s12896-021-00695-6

**Published:** 2021-05-17

**Authors:** S. Sloan, C. J. Jenvey, D. Piedrafita, S. Preston, M. J. Stear

**Affiliations:** 1 AgriBio Centre for AgriBioscience, Department of Animal, Plant and Soil Sciences, School of Life Sciences, La Trobe University, 5 Ring Road, Bundoora, Victoria 3086 Australia; 2 School of Science, Psychology and Sport, Federation University, Churchill, Victoria Australia

**Keywords:** DNA isolation, Teladorsagia circumcincta, DNA extraction, Polymerase chain reaction, Nematode, Genome sequencing, Methodology

## Abstract

**Background:**

The purpose of this study was to develop a reliable DNA extraction protocol to use on individual *Teladorsagia circumcincta* nematode specimens to produce high quality DNA for genome sequencing and phylogenetic analysis. Pooled samples have been critical in providing the groundwork for *T. circumcincta* genome construction, but there is currently no standard method for extracting high-quality DNA from individual nematodes. 11 extraction kits were compared based on DNA quality, yield, and processing time.

**Results:**

11 extraction protocols were compared, and the concentration and purity of the extracted DNA was quantified. Median DNA concentration among all methods measured on NanoDrop 2000™ ranged between 0.45–11.5 ng/μL, and on Qubit™ ranged between undetectable – 0.962 ng/μL. Median A260/280 ranged between 0.505–3.925, and median A260/230 ranged − 0.005 – 1.545. Larval exsheathment to remove the nematode cuticle negatively impacted DNA concentration and purity.

**Conclusions:**

A *Schistosoma sp.* DNA extraction method was determined as most suitable for individual *T. circumcincta* nematode specimens due to its resulting DNA concentration, purity, and relatively fast processing time.

**Supplementary Information:**

The online version contains supplementary material available at 10.1186/s12896-021-00695-6.

## Background

Parasitic infections of livestock are of major socio-economic importance worldwide. Internal parasites of sheep alone have been shown to cost $436 million annually in Australia [1]. Therefore, major economic gains are to be made by improving the control of parasitic diseases. Parasitic infection of livestock is largely controlled by anthelmintic treatment, however, drug resistance is rapidly developing [2–4]. Additional methods of control include nutritional supplementation, vaccination, selective breeding, and pasture management, which are used with varying success [5–9].

*Teladorsagia circumcincta* is the most important parasitic nematode of sheep in cool temperate regions worldwide [3]. Clinical disease cause by *T. circumcincta* infection results in reduced production, decreased animal welfare, parasitic gastroenteritis, poor growth performance, and weight loss [10]. To develop new control strategies for *T. circumcincta* infection, it is pivotal that research uncovers as much information about the biology of this nematode as possible. An important starting point involves the genomic investigation of *T. circumcincta*.

Nematode species are genetically diverse and identification of variation in genes amongst populations requires the analysis of individual nematodes. Effective extraction of high-quality DNA from individual specimens is essential to assist in developing improved diagnostic methods. *T. circumcincta* are slender, reddish-brown worms with a short buccal cavity [11]. Their size varies considerably amongst sheep, as worm length is affected by host immune pressure [12], but typically female size ranges from 8 to 10 mm, and males 6–8 mm [11]. Due to their small size, there is very limited tissue from which to derive genetic material. Pooled samples have been critical in providing the groundwork for *T. circumcincta* genome construction [13–15], but there is currently no standard method for extracting high-quality DNA from individual nematodes.

With so many commercially available extraction kits on the market there is a need to systematically compare the different methods for optimal extraction of *T. circumcincta* DNA. The method must ensure that adequate *T. circumcincta* DNA is being extracted, as internal parasites are likely to harbour host and host microorganism DNA, in addition to their own, causing complications downstream. The ideal extraction method should optimise DNA quantity, avoid contamination, minimise degradation and inhibitors, require low-cost consumables and equipment, and have rapid processing time. The quantity and purity of DNA are also important for downstream applications, such as PCR or genome sequencing.

Studies focusing on several DNA extraction methods are uncommon. SR Doyle et al. [16] compared 5 methods to extract individual nematode DNA from 8 different species and found a Cancer Genome Project method was ideal. This method utilised Whatman® FTA® cards for sample collection which did not limit the DNA extraction and whole genome sequencing of parasite samples. Y Seesao et al. [17] compared 4 methods for extraction of pooled Anisakidae nematodes and found a silica binding column was the best method because it provided good quality and quantity DNA repeatedly and at low cost, however, modifications to the protocols to breakdown the complex nematode cuticle was required. LM Schiebelhut et al. [18] compared 8 extraction techniques for species comprising 8 separate phyla and found silica binding column methods produced quality DNA quickly, but commercial kits are costly. RL Smith et al. [19] compared 13 extraction methods for ancient powdery mildew specimens and found a silica binding column method was most suitable and that DNA concentration was more important than quality for whole genome next generation sequencing purposes on limited and valuable specimens.

Three types of DNA extraction have been tested in this study: chelating, precipitation, and silica binding. Chelex™ is a chelating ion-exchange resin that binds polar components of cells leading to disruption of cell membranes, cell lysis and denaturation of DNA. The remaining non-polar DNA is retained in the aqueous solution above the Chelex™ [20, 21]. Precipitation extraction involves salt and ethanol added to an aqueous solution which precipitates nucleic acids. Silica binding methods bind DNA to silica surfaces in the presence of certain salts and under certain pH conditions [22]. All three methods have their place in DNA extraction, with some working better than others in different circumstances, and it is a comparison of various methods that determines which type is most appropriate for a particular species or sample type.

Here we have compared multiple DNA extraction protocols to determine whether silica binding column, precipitation or chelating methods are most suitable for individual *T. circumcincta* nematode DNA extraction. The aim of this study was to compare 11 different DNA extraction protocols on individual *T. circumcincta* nematode specimens based on the yield, quality and reliability of DNA, and protocol time, to obtain DNA suitable for use in PCR and genome sequencing applications.

## Results

11 common DNA extraction protocols were compared and selected to encompass a range of extraction methods and modifications, as well as prior availability in the laboratory. These methods included AccuPrep® Genomic DNA Extraction - Mammalian Tissue (AccM), AccuPrep® Genomic DNA Extraction Kit – Whole Blood, Buffy Coat and Cultured Cells (AccW), Chelex®100 (CheX), cetyl trimethyl ammonium bromide (CTAB), E.Z.N.A.® Forensic DNA (EznF), Isolate II Genomic DNA Kit (IsoG), *Schistosoma sp.* DNA Extraction Method (Schi), Schi with larval exsheathment (Schi-LE), sodium dodecyl sulphate (SDS), Wizard® Genomic DNA Purification Kit - Mouse Tail (WizM), and Wizard® Genomic DNA Purification - Plant Tissue (WizP) (Table 1). These protocols were compared based on DNA concentration, quality, purity, and protocol time. The DNA samples were expected to comprise *T. circumcincta* DNA, host DNA from sheep, as well as DNA from microorganisms present in the sheep gut prior to nematode collection. PCR and ITS-2 phylogeny were performed to confirm the protocols would extract *T. circumcincta* DNA.

**Table 1:** DNA extraction protocols tested on six individual adult, female *T. circumcincta* specimens per method in this study

Method or kit name	Protocol code	Reference or supplier (catalogue #)	Extraction method	Time required (hours)
AccuPrep® Genomic DNA Extraction - Mammalian Tissue	AccM	Bioneer (K-3032)	Silica binding	1.5
AccuPrep® Genomic DNA Extraction Kit - Whole Blood, Buffy Coat and Cultured Cells	AccW	Bioneer (K-3032)	Silica binding	0.5
Chelex®100	CheX	PS Walsh et al. [20]	Chelating	0.5
Cetyl Trimethyl Ammonium Bromide	CTAB	T Sarkinen et al. [23]	Precipitation	6
E.Z.N.A.® Forensic DNA	EznF	Omega Bio-tek (D3591–00)	Silica binding	1.5–2
Isolate II Genomic DNA Kit	IsoG	Bioline (BIO-52066)	Silica binding	1.5–3
*Schistosoma sp.* DNA Extraction Method	Schi	PJ Brindley et al. [24]	Precipitation	1.5
Sodium Dodecyl Sulphate	SDS	K Edwards et al. [25]	Precipitation	1.25–1.75
Wizard® Genomic DNA Purification Kit - Mouse Tail	WizM	Promega (A1120)	Precipitation	4–4.5^a^
Wizard® Genomic DNA Purification - Plant Tissue	WizP	Promega (A1120)	Precipitation	1^a^
Larval exsheathment	-LE	HJ Dawkins et al. [26]	–	1

^a^: Additional overnight incubation required

### DNA concentration

**Fig. 1:**
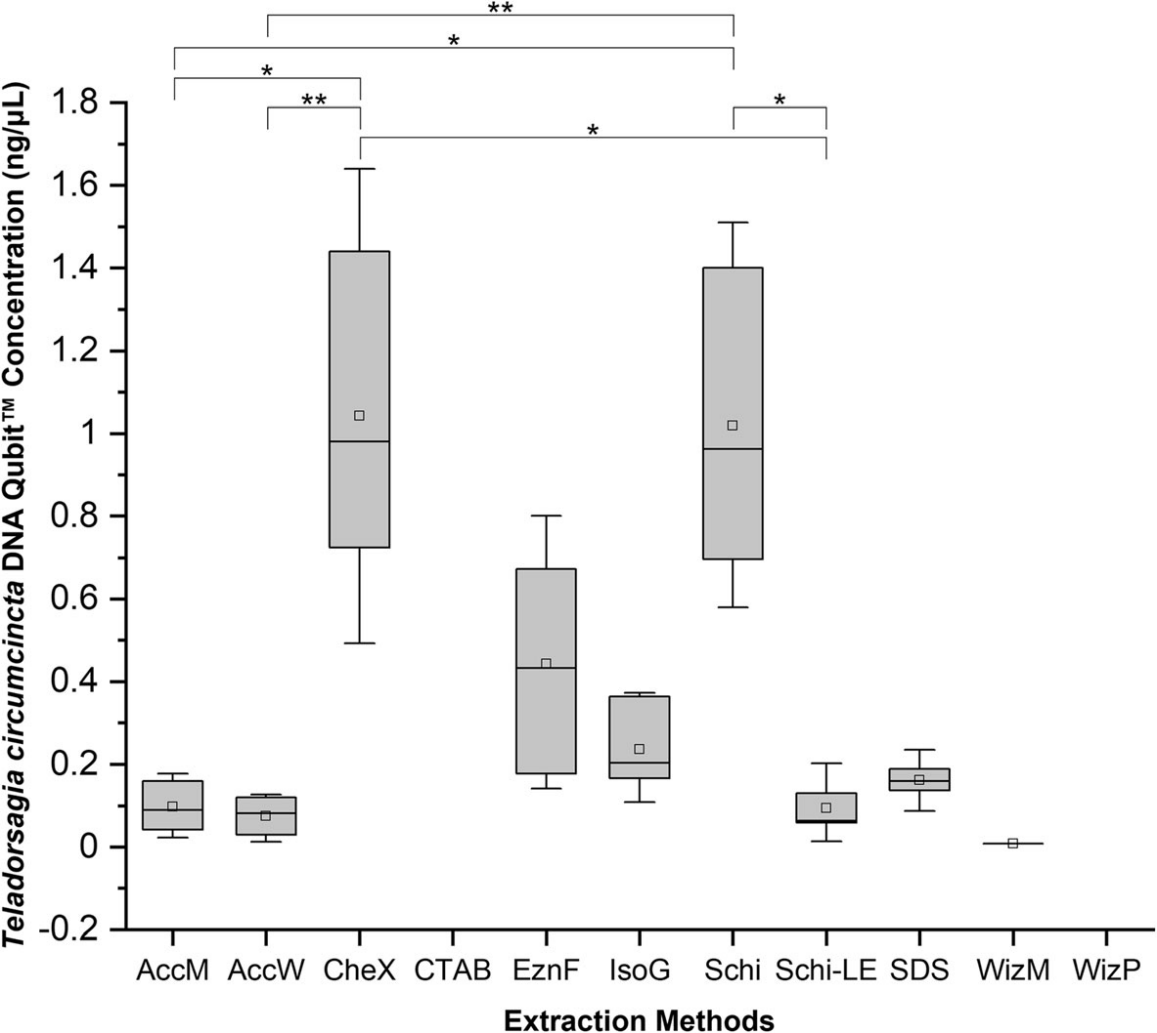
Box-and-whisker plots of the DNA concentration (ng/μL) measured by Invitrogen Qubit™ fluorometer. 11 DNA extraction protocols were used, each with 6 individual adult, female *T. circumcincta* specimens. Boxes extend from the 25th to the 75th percentile of each group’s distribution of values; Whiskers above and below the box indicate the 10th and 90th percentiles. Median line ^__^; Mean □; Dunn’s Multiple Comparison test significance between methods indicated by asterisk; *: 0.01 < *p* < 0.05; **: 0.001 < *p* < 0.01. Extraction method abbreviations: AccM (AccuPrep® Genomic DNA Extraction - Mammalian Tissue), AccW (AccuPrep® Genomic DNA Extraction Kit – Whole Blood, Buffy Coat and Cultured Cells), CheX (Chelex®100), CTAB (cetyl trimethyl ammonium bromide), EznF (E.Z.N.A.® Forensic DNA), IsoG (Isolate II Genomic DNA Kit), Schi (*Schistosoma sp.* DNA Extraction Method), Schi-LE (Schi with larval exsheathment), SDS (sodium dodecyl sulphate), WizM (Wizard® Genomic DNA Purification Kit - Mouse Tail), WizP (Wizard® Genomic DNA Purification - Plant Tissue)

**Fig. 2:**
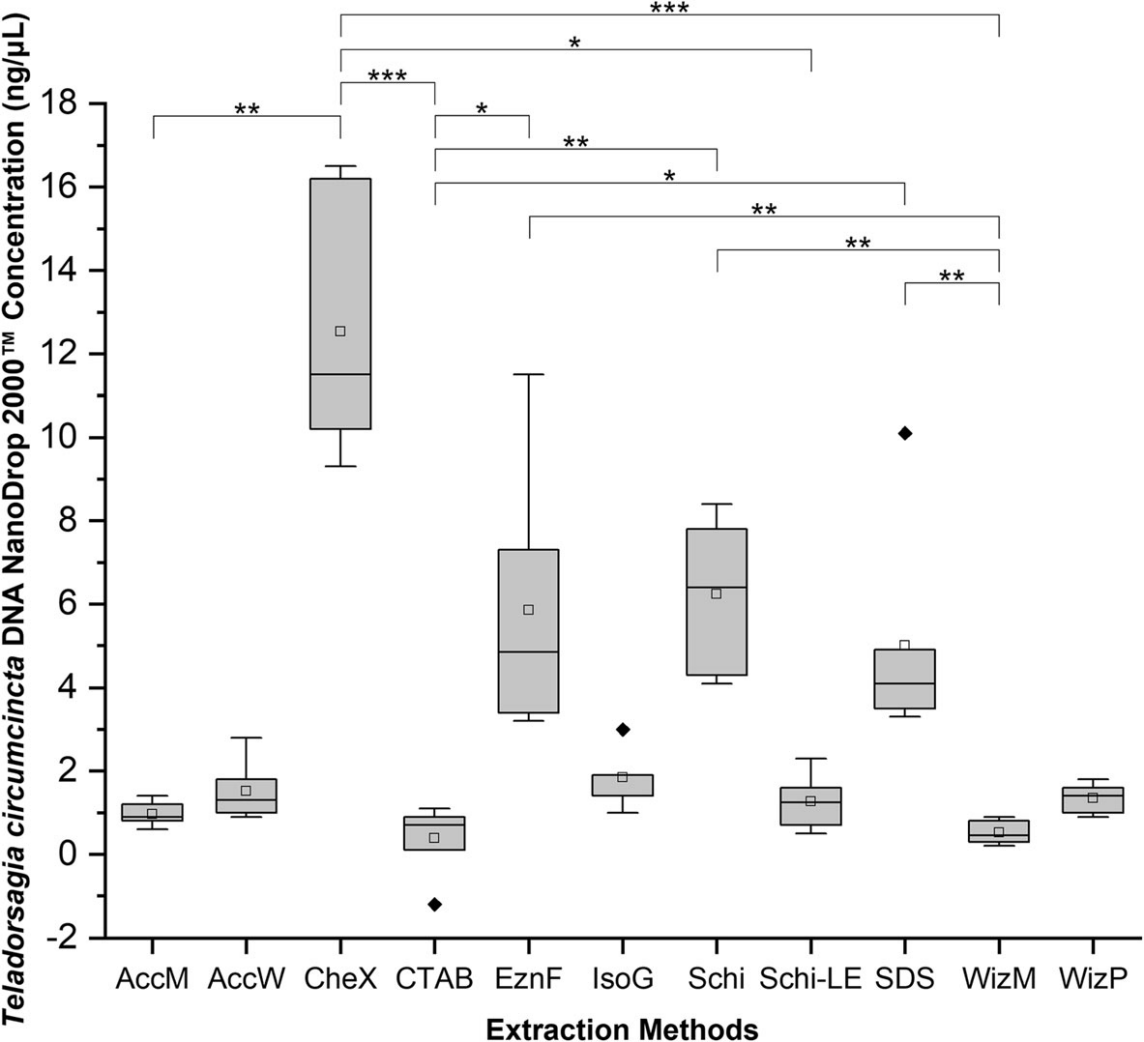
Box-and-whisker plots of the DNA concentration (ng/μL) measured by ThermoScientific NanoDrop 2000™. 11 DNA extraction protocols were used, each with 6 individual adult, female *T. circumcincta* specimens. Boxes extend from the 25th to the 75th percentile of each group’s distribution of values; Whiskers above and below the box indicate the 10th and 90th percentiles. Median line ^__^; Mean □; Outliers ♦; Dunn’s Multiple Comparison test significance between methods indicated by asterisk; *: 0.01 < *p* < 0.05; **: 0.001 < *p* < 0.01; ***: *p* < 0.001. Extraction method abbreviations: AccM (AccuPrep® Genomic DNA Extraction - Mammalian Tissue), AccW (AccuPrep® Genomic DNA Extraction Kit – Whole Blood, Buffy Coat and Cultured Cells), CheX (Chelex®100), CTAB (cetyl trimethyl ammonium bromide), EznF (E.Z.N.A.® Forensic DNA), IsoG (Isolate II Genomic DNA Kit), Schi (*Schistosoma sp.* DNA Extraction Method), Schi-LE (Schi with larval exsheathment), SDS (sodium dodecyl sulphate), WizM (Wizard® Genomic DNA Purification Kit - Mouse Tail), WizP (Wizard® Genomic DNA Purification - Plant Tissue)

The 11 different DNA extraction protocols generated variable concentrations of DNA from *T. circumcincta* nematode specimens (Table 2). The Qubit™ fluorometer and NanoDrop 2000™ spectrophotometer produced different readings of DNA concentration. The Qubit™ consistently estimated lower concentrations compared to the NanoDrop 2000™. Based on Qubit™ fluorometer quantification, the CheX protocol produced the highest DNA concentration (0.98 ng/μL), followed by Schi (0.962 ng/μL) and EznF (0.4325 ng/μL) (Fig. 1). The remaining protocols produced DNA concentrations < 0.3 ng/μL, with WizP and CTAB at undetectable levels. Concentrations assessed with the NanoDrop 2000™ spectrophotometer followed a similar pattern, with CheX, Schi and EznF showing the highest median DNA concentrations of 11.5 ng/μL, 6.4 ng/μL and 4.85 ng/μL, respectively. The remaining protocols had readings higher than their Qubit™ counterparts, ranging between 0.45–4.1 ng/μL (Fig. 2).

**Table 2:** Median DNA yield (ng/μL), total DNA yield (ng), and quality (A260/280 and A260/230) of 11 extraction protocols tested on 6 individual adult, female *Teladorsagia circumcincta* specimens

Extraction Method	Median (Range) Invitrogen Qubit™ DNA Concentration (ng/μl)	Median (Range) Invitrogen Qubit™ DNA Total Yield (ng)	Median (Range) NanoDrop 2000™ DNA Concentration (ng/μl)	Median (Range) NanoDrop 2000™ DNA Total Yield (ng)	Median (Range) NanoDrop 2000™ DNA Quality (A260/280)	Median (Range) NanoDrop 2000™ DNA Quality (A260/230)
AccM	0.0897 (0.0228–0.178)	4.485 (2.12–8.9)	0.9 (0.6–1.4)	45 (30–70)	0.935 (−13.1–1.64)	0.53 (0.18–0.73)
AccW	0.08125 (0.0129–0.127)	4.0625 (0.645–6.35)	1.3 (0.9–2.8)	65 (45–140)	1.73 (− 5.76–3.58)	0.77 (0.62–1.52)
CheX	0.98 (0.429–1.64)	49 (24.6–82)	11.5 (9.3–16.5)	575 (465–825)	1.99 (1.63–2.37)	0.535 (0.46–0.67)
CTAB	Undetectable	Undetectable	0.7 (−1.2–1.1)	35 (− 60–55)	0.675 (− 13.83–1.51)	0.395 (−1.02–0.98)
EznF	0.4325 (0.142–0.672)	21.625 (7.1–40)	4.85 (3.2–11.5)	242.5 (160–575)	2.375 (1.99–2.83)	1.305 (0.84–2.34)
IsoG	0.2035 (0.109–0.372)	10.175 (5.45–18.15)	1.9 (1.0–3.0)	95 (50–150)	1.87 (− 24.56–3.27)	1.545 (0.52–3.51)
Schi	0.962 (0.58–1.51)	48.1 (29–75.7)	6.4 (4.1–8.4)	320 (205–420)	2.19 (1.68–2.77)	0.99 (0.46–2.77)
Schi-LE	0.064 (undetectable – 0.203)	3.08 (undetectable – 10.15)	1.25 (0.5–2.3)	62.5 (25–115)	2.12 (− 6.04–5.39)	0.245 (0.08–0.54)
SDS	0.16 (undetectable – 0.236)	7.425 (undetectable – 11.8)	4.1 (3.3–10.1)	205 (165–505)	3.925 (− 33.69–10.76)	0.05 (0.03–0.11)
WizM	0.0079 (undetectable – 0.0079)	0 (0–0.395)	0.45 (0.2–0.9)	22.5 (10–45)	0.985 (− 3.65–3.78)	− 0.005 (− 1.4–0.42)
WizP	Undetectable	Undetectable	1.4 (0.9–1.8)	70 (45–90)	0.505 (− 12.31–17.77)	0.465 (0.26–1.06)

A Kruskal-Wallis non-parametric test indicated significant differences in DNA concentration between the methods investigated for NanoDrop 2000™ (χ^2^ = 55.821, *P* = 2.218e-08) and Qubit™ (χ^2^ = 36.148, *P* = 1.65e-05). A post-hoc Dunn’s Multiple Comparison Test was conducted to determine significant pairwise differences between methods. Significant differences were found between several methods in NanoDrop 2000™, and Qubit™ DNA concentration. Exsheathment prior to DNA extraction was found to reduce the concentration of DNA extracted by both NanoDrop 2000™ and Qubit™.

### DNA purity

**Fig. 3:**
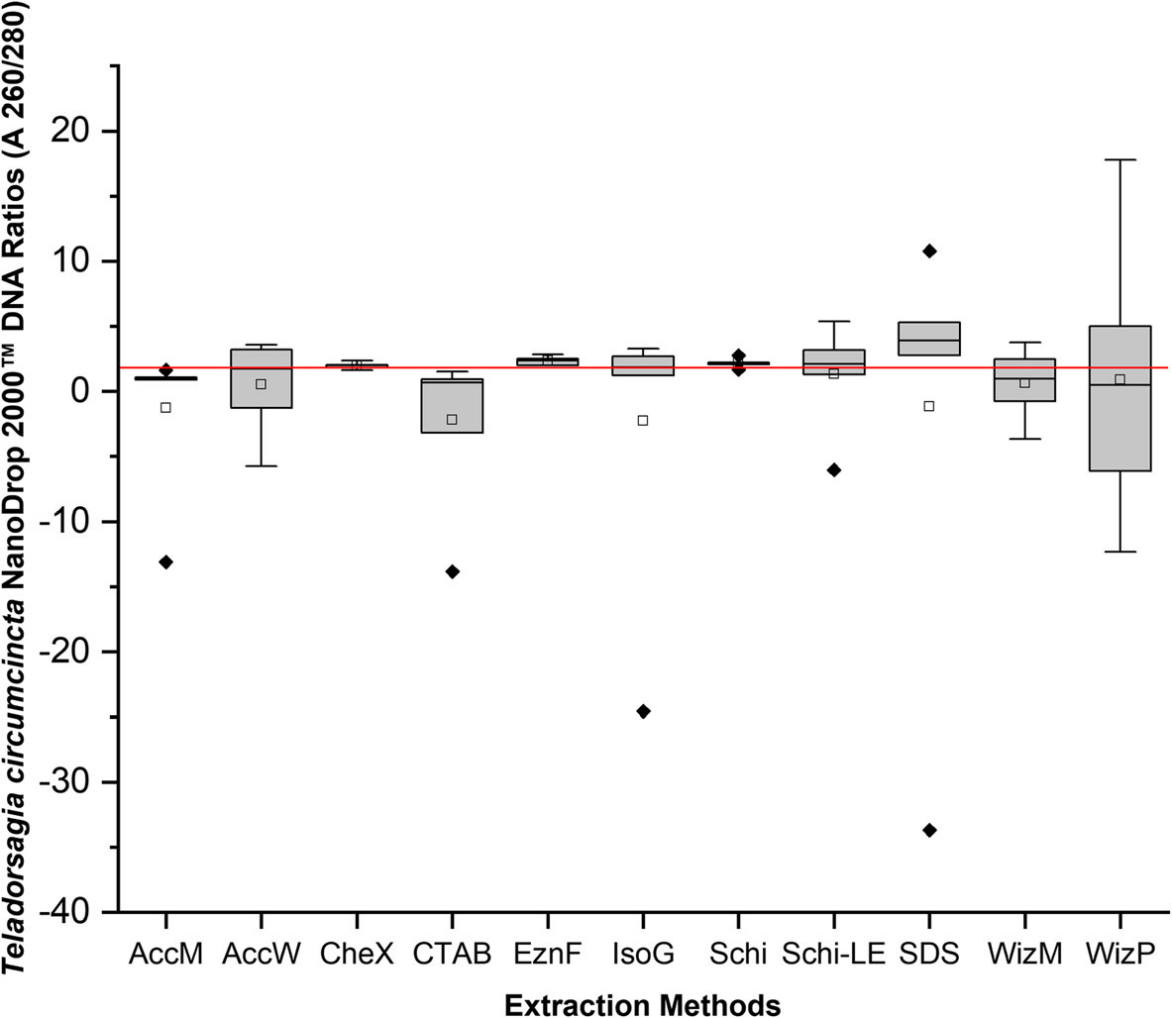
Box-and-whisker plots of DNA quality measured by ThermoScientific NanoDrop 2000™ absorbency measurement 260 nm / 280 nm ratios. 11 DNA extraction protocols were used, each with 6 individual adult, female *T. circumcincta* specimens. Boxes extend from the 25th to the 75th percentile of each group’s distribution of values; Whiskers above and below the box indicate the 10th and 90th percentiles. The red line indicates the desired absorbency ratio of 1.8. Median line ^__^; Mean □; Outliers ♦. Extraction method abbreviations: AccM (AccuPrep® Genomic DNA Extraction - Mammalian Tissue), AccW (AccuPrep® Genomic DNA Extraction Kit – Whole Blood, Buffy Coat and Cultured Cells), CheX (Chelex®100), CTAB (cetyl trimethyl ammonium bromide), EznF (E.Z.N.A.® Forensic DNA), IsoG (Isolate II Genomic DNA Kit), Schi (*Schistosoma sp.* DNA Extraction Method), Schi-LE (Schi with larval exsheathment), SDS (sodium dodecyl sulphate), WizM (Wizard® Genomic DNA Purification Kit - Mouse Tail), WizP (Wizard® Genomic DNA Purification - Plant Tissue)

**Fig. 4:**
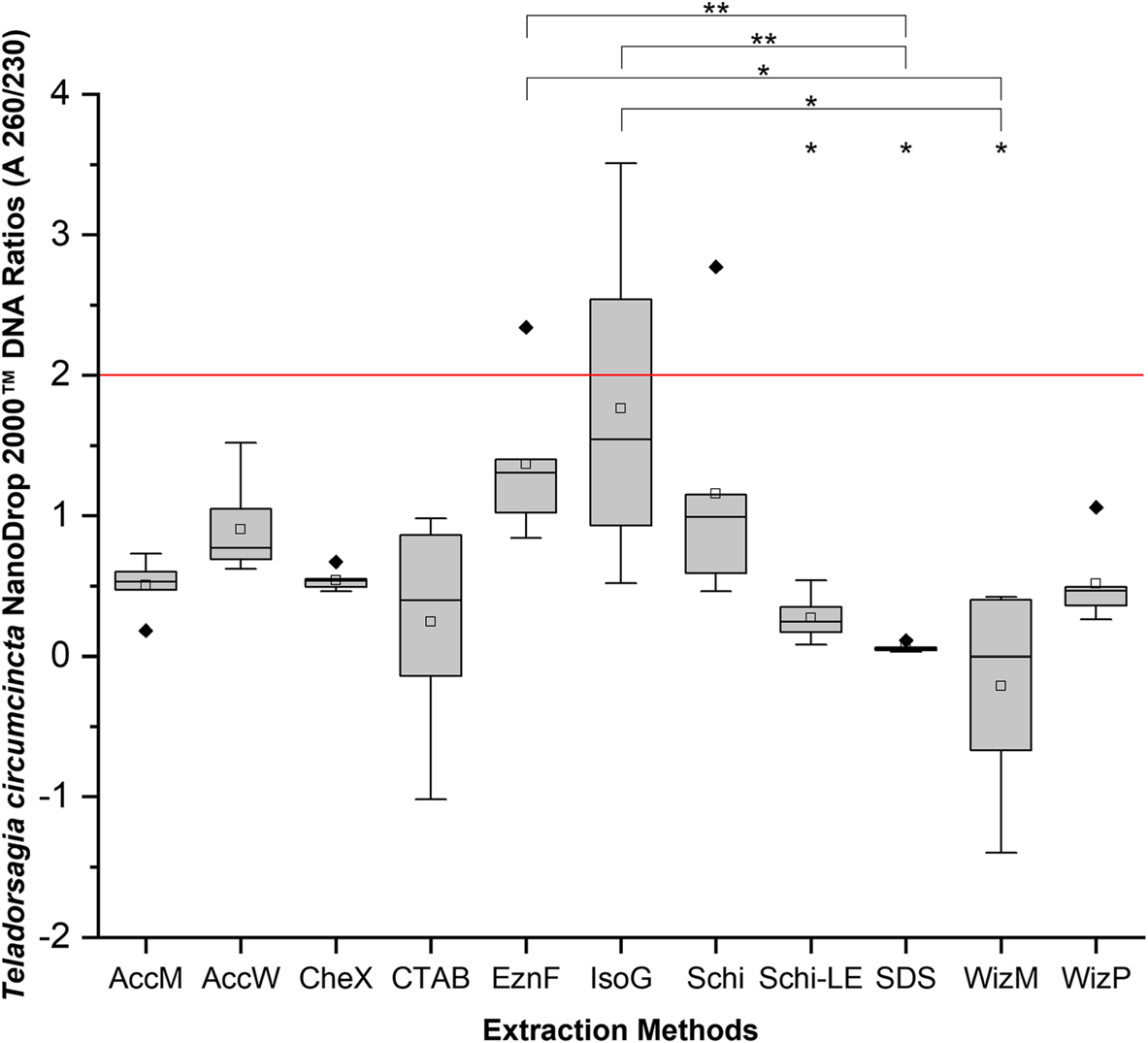
Box-and-whisker plots of DNA quality measured by ThermoScientific NanoDrop 2000™ absorbency measurement 260 nm / 230 nm ratios. 11 DNA extraction protocols were used, each with 6 individual adult, female *T. circumcincta* specimens. Boxes extend from the 25th to the 75th percentile of each group’s distribution of values; Whiskers above and below the box indicate the 10th and 90th percentiles. The red line indicates the desired absorbency ratio of 2.0. Median line ^__^; Mean □; Outliers ♦; Dunn’s Multiple Comparison test significance between methods indicated by asterisk; *: 0.01 < p < 0.05; **: 0.001 < p < 0.01; ***: 0.0001 < p < 0.001. Extraction method abbreviations: AccM (AccuPrep® Genomic DNA Extraction - Mammalian Tissue), AccW (AccuPrep® Genomic DNA Extraction Kit – Whole Blood, Buffy Coat and Cultured Cells), CheX (Chelex®100), CTAB (cetyl trimethyl ammonium bromide), EznF (E.Z.N.A.® Forensic DNA), IsoG (Isolate II Genomic DNA Kit), Schi (*Schistosoma sp.* DNA Extraction Method), Schi-LE (Schi with larval exsheathment), SDS (sodium dodecyl sulphate), WizM (Wizard® Genomic DNA Purification Kit - Mouse Tail), WizP (Wizard® Genomic DNA Purification - Plant Tissue)

DNA purity was measured using 260 nm / 280 nm ratio (A260/280) and 260 nm / 230 nm ratio (A260/230) NanoDrop 2000™ spectrophotometer absorbency measurements. The optimal values indicating high quality DNA are 1.8 and 2.0, respectively. Most methods produced DNA with an A260/280 ranging between 1.7–2.4, with CheX, Schi and EznF methods ranging most consistently to the optimal value (Table 2, Fig. 3). IsoG was the only method whose A260/230 range met the 2.0 target value, however, the median value fell short at 1.545, indicating organic contaminants. All remaining methods ranged from − 0.005 – 1.305 (Fig. 4).

A Kruskal-Wallis non-parametric test indicated significant differences in DNA purity between the methods investigated for A260/280 (χ^2^ = 21.102, *P* = 0.03233), however, a post-hoc Dunn’s Multiple Comparison Test determined there were no significant pairwise differences between methods in the A260/280 measurements (Additional File 1). The Kruskal-Wallis test indicated significant differences in DNA purity between the methods investigated for A260/230 (χ^2^ = 52.979, *P* = 1.811e-07). Only Schi-LE, SDS and WizM were significantly different from the optimal A260/230 value (Fig. 4). Silica-binding column methods had higher quality extractions, while precipitation methods had greater DNA yield. Schi and Schi-LE indicate larval exsheathment negatively affects purity of DNA obtained as indicated by A260/280 and A260/230 analyses (Figs. 3, 4).

### PCR amplification of isolated DNA

**Fig. 5:**
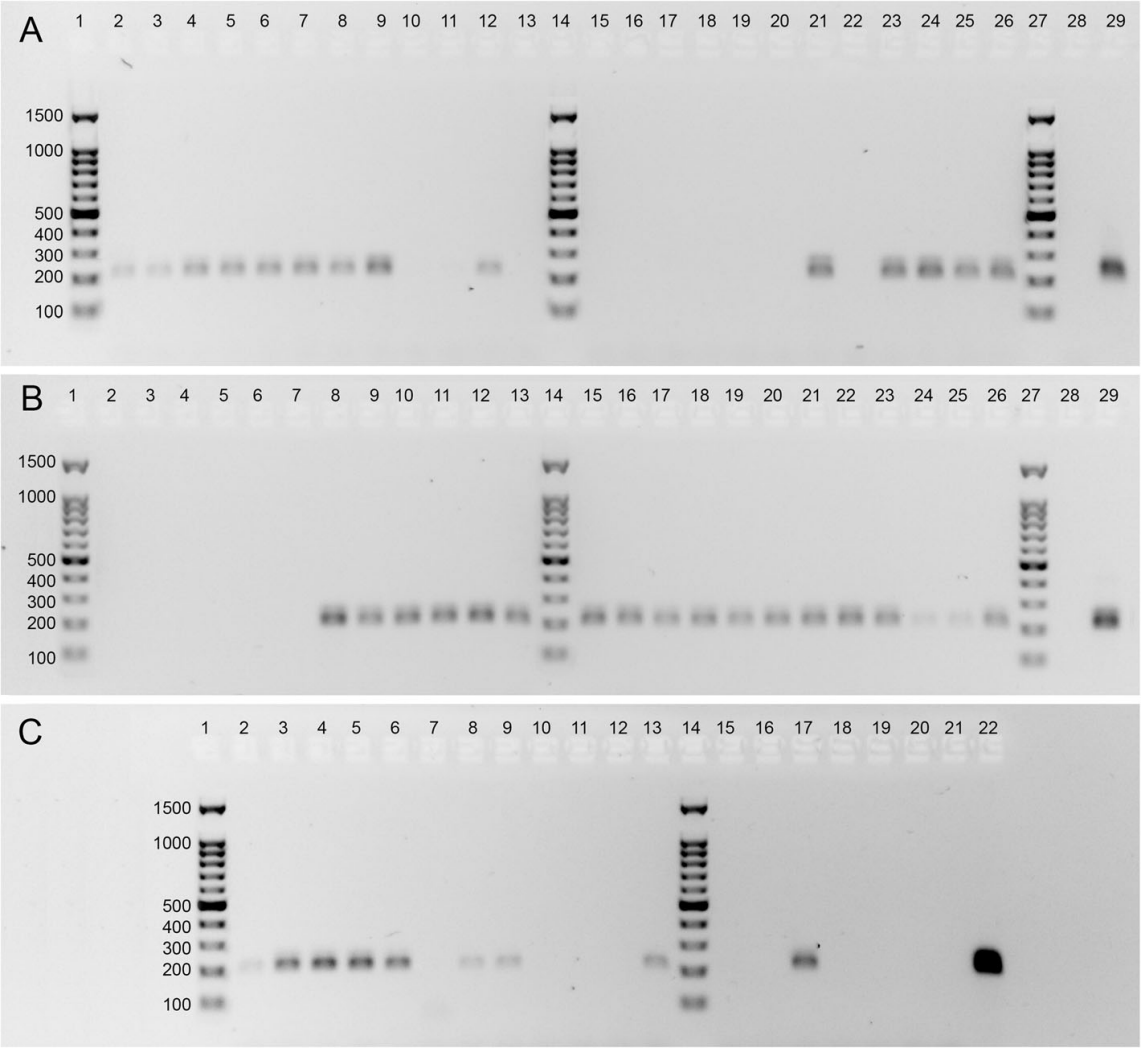
Agarose gels displaying amplicons produced by conventional PCR using primer pair ITS-2/NC2. Genomic DNA samples from single *Teladorsagia circumcincta* nematode specimens. Ladder indicating amplicon size (bp) (lanes 1, 14, 27). **a** Schi (lanes 2–7), Schi-LE (lanes 8–13), SDS (lanes 15–20), CheX (lanes 21–26), no-DNA control (lane 28), *T. circumcincta* ITS-2 gBlocks™ Gene Fragment (Integrated DNA Technologies) control (lane 29). **b** WizP (lanes 2–7), EznF (lanes 8–13), AccM (lanes 15–20), IsoG (lanes 21–26), no-DNA control (lane 28), *T. circumcincta* ITS-2 gBlocks™ Gene Fragment control (lane 29). C: AccW (lanes 2–7), WizM (lanes 8–13), CTAB (lanes 15–20), no-DNA control (lane 21), *T. circumcincta* ITS-2 gBlocks™ Gene Fragment control (lane 22). The specificity of individual amplicons produced was verified by direct sequencing

PCR success was indicated by the presence of a visible band at the expected target size of 219 bp for each sample on a 1% agarose electrophoresis gel (Fig. 5), corresponding to the *T. circumcincta* ITS-2 region. Overall, 41/66 (62.1%) of the DNA extractions were positive for *T. circumcincta* ITS-2 DNA. Six methods were consistently able to extract *T. circumcincta* DNA, with AccM, EznF, IsoG and Schi as the most consistent (6/6 samples), and AccW and CheX slightly less (5/6). Schi-LE and WizM were able to extract *T. circumcincta* DNA half of the time, while SDS and WizP were not able to extract *T. circumcincta* DNA (Table 3). The chelating method was able to extract *T. circumcincta* DNA most of the time (5/6). Three of the silica binding column methods were 100% successful at PCR amplicon amplification, and 1 was 83.3% successful. Precipitation methods were variable, 1 was 100% successful (Schi) while others were successful in 50% of samples (Schi-LE and WizM) or less (CTAB, SDS and WizP) (Table 3). Larval exsheathment greatly reduced the reliability of DNA extraction. Four samples (CTAB 3, and WizM 1, 2, 6) which had undetectable DNA concentration when measured on Qubit™ fluorometer were positive for ITS-2 amplification (Fig. 5c).

**Table 3:** Successful amplicon, sequencing, and median amplicon length of 11 extraction protocols tested on 6 individual adult, female *Teladorsagia circumcincta* specimens

Method	Successful Amplicon	Successful Amplicon Sequencing	Median Sequence Length (bp) (Range)
AccM	6/6 (100%)	1/6 (16.7%)	93
AccW	5/6 (83.3%)	1/5 (20%)	84
CheX	5/6 (83.3%)	1/5 (20%)	53
CTAB	1/6 (16.7%)	1/1 (100%)	84
EznF	6/6 (100%)	3/6 (50%)	72 (58–78)
IsoG	6/6 (100%)	3/6 (50%)	84 (53–183)
Schi	6/6 (100%)	1/6 (16.7%)	84
Schi-LE	3/6 (50%)	2/3 (66.7%)	135 (84–186)
SDS	0/6 (0%)	–	–
WizM	3/6 (50%)	1/3 (33.3%)	67
WizP	0/6 (0%)	–	–

All samples showing a positive amplification on the PCR agarose gel were sequenced. Sequencing of the approximately 219 bp fragment confirmed that 14/41 PCR products were from our target organism. 27/41 obtained fragments produced overlays (multiple peaks) and degradation of DNA was detected. 10/27 obtained fragments were below the Q20 quality cut-off. The 14 sequences which passed QC are available at GenBank (accession numbers MW161470–MW161483).

### Sequence analysis

Fourteen ITS-2 sequences (Tc 1–14) from *T. circumcincta* were obtained from 9 of the DNA extraction protocols. AccM, AccW, CheX, CTAB, Schi, and WizM produced 1 sequence each, Schi-LE produced 2 sequences, and EznF and IsoG each produced 3 sequences.

**Fig. 6:**
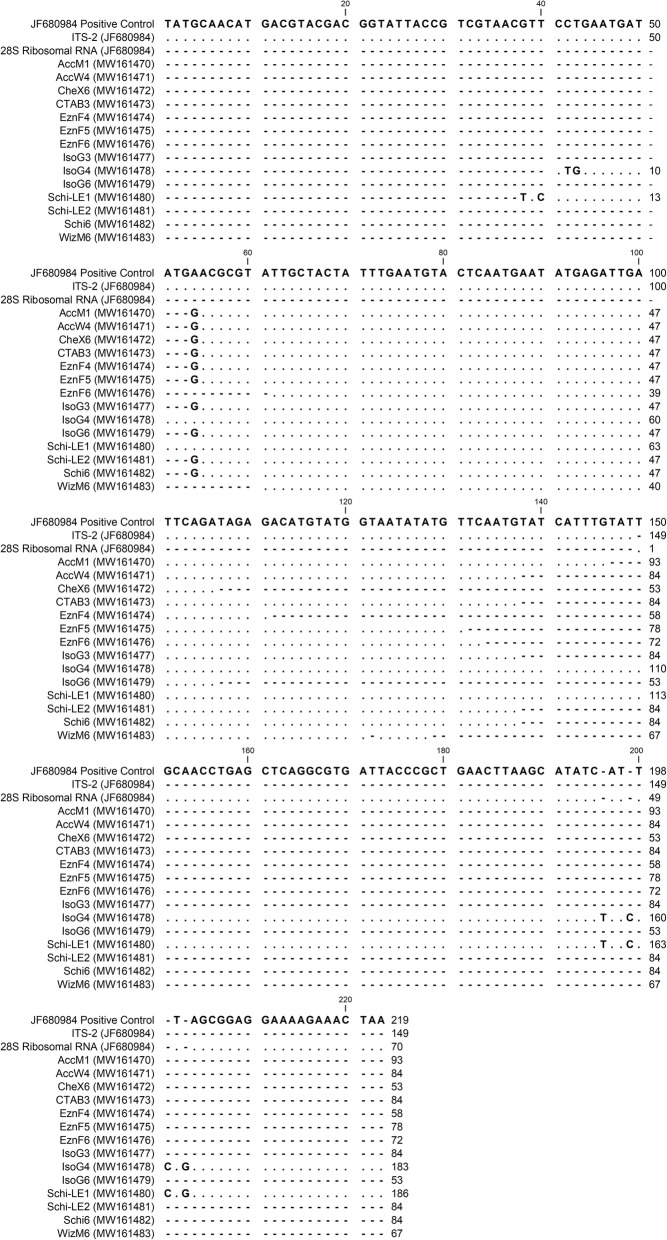
Multiple sequence alignment of *Teladorsagia circumcincta* ITS-2 PCR products. PCR products (GenBank accessions MW161470-MW161483) and the positive control reference (GenBank accession JF680984) include ITS-2 and 28S ribosomal RNA genes. Conserved residues indicated by a dot, gaps indicated by a dash

Fragment lengths obtained for Tc 1–14 ranged from 53 to 186 bp. The sequences were mostly identical when aligned against the positive control reference (Fig. 6) and included segments of both ITS-2 and 28S ribosomal RNA genes. Small differences between the sequences were observed; AccM1, AccW4, CheX6, CTAB3, EznF4 and 5, IsoG3 and 6, Schi-LE2, and Schi6 substituted in base G at position 54. Additionally, WizM6 had one missing base (T) at position 122, and IsoG4 and Schi-LE1 had four additional bases (T, C, C, G) at positions 196, 199, 201 and 203, respectively. Whether these differences were due to variation in the gene or sequencing error was not determined.

## Discussion

This is the first systematic comparison of different DNA extraction methods for individual *T. circumcincta* nematodes. The three types of DNA extraction tested in this study were chelating, precipitation, and silica binding. DNA extraction method comparisons have previously been conducted on individual nematodes of other species [16], however, they did not include the traditional phenol-chloroform/CTAB method, or a chelating method as has been done in this study. The variety of DNA extraction methods available highlights the difficulty in comparing and standardizing methods between studies and organisms.

Comparison of DNA extraction methods is necessary to determine which is ideal for *T. circumcincta*. Overall, in this study silica binding column extraction methods had greater quality of *T. circumcincta* DNA, while precipitation methods were superior in terms of total DNA yield. Precipitation methods have been used on individual *T. circumcincta* specimens [27, 28], and the current reference genome for *T. circumcincta* [15]. However, other studies on *T. circumcincta,* whether pooled samples or individual specimens, have used silica binding columns [29–31]. There is currently no consensus on which DNA extraction method is best for *T. circumcincta*.

Of the 11 extraction methods compared in this study, four had relatively higher DNA concentration and purity; CheX, EznF, IsoG and Schi, which represent all three extraction types. The processing time for these methods was on the longer end of the spectrum but were relatively short compared to methods used in other studies which have exceeded 12 h [15, 31]. The length of time needed to complete each method could be a factor determining method choice. The longer the method takes, and more hands-on the steps, determines the feasibility of a laboratory to carry out the method, and how many samples can be processed at a time or in a day. CheX was the shortest method of all, barely exceeding 30 min from start to finish. EznF, IsoG and Schi took 1.5–3 h to complete depending on the method and were more manually intensive. Schi and CheX were consistently superior in terms of DNA quantity and quality, and processing time.

Comparing quantitative and qualitative data is valuable in understanding the DNA extraction output. NanoDrop 2000™ and Qubit™ have their limitations but when used together can be powerful tools. Previous studies have claimed that spectrophotometry is the better measurement for DNA quantification [32], while most favour fluorometry [19, 33–35].

Qubit™ is based on fluorometric analysis. A fluorescent dye binds specifically to the nucleic acids within a sample and the DNA is quantified by the fluorescence measured by the detector. Even if the sample is contaminated, it can give an accurate reading because the dye is bound only to DNA. Qubit™ is highly regarded for use in sequencing and PCR because it can quantify DNA as low as 10 pg – 200 ng. The Qubit™ is considered a very accurate quantification method, more so than the NanoDrop 2000™, however, an additional instrument is required to measure the quality of a sample [36]. This study used the NanoDrop 2000™ for this additional qualitative analysis.

NanoDrop 2000™ analyses spectrophotometric absorbance. DNA absorbs light at 260 nm, however, it does not distinguish between double- and single-stranded DNA, RNA, and nucleotides. Furthermore, impurities such as protein, phenol and other salts may also measure readings at this wavelength. To account for this, the purity of DNA relative to contaminants can be determined by measuring the ratio of different wavelengths, for example, A260/280 and A260/230 [37]. The A260/280 ratio is used to determine the presence of protein in a sample, and a pure DNA A260/280 ratio should be 1.8. Lower ratios indicate protein contamination. The A260/230 ratio is used to indicate the presence of organic contaminants which could affect downstream applications. A pure DNA sample should have an A260/230 ratio of 2.0. These two ratios are used to determine the purity of a DNA sample. NanoDrop 2000™ is not as sensitive as Qubit™, quantifying 10 ng – 10 μg [37]. Additionally, calibration of the instruments is crucial to ensure the readings are as accurate as possible.

CheX, Schi and EznF had a tight IQR over the optimal A260/280 value of 1.8 indicating few contaminating proteins. However, negative A260/280 ratios were determined for several samples (Table 2). The NanoDrop 2000™ was blanked with the elution buffer of the respective method, and a negative ratio would mean that one of the wavelengths is absorbing less than zero light. This is indicative of insoluble contamination on the NanoDrop 2000™ lens. The lens was cleaned thoroughly between readings, so it could be that an insoluble contaminant has eluted with the DNA sample and caused inaccurate readings, or it is user error. The negative A260/280 readings were not exclusive to a method type, it was observed in both silica binding and precipitation methods. 3/4 methods of choice (CheX, EznF, and Schi) did not result in any negative A260/280 readings, further confirming their superiority amongst the methods compared in this study whether because the method does not allow, or user error does not result in, insoluble contaminants. The decision to retain these samples and results, and not do a new round of extractions was to show what results are possible with each of these methods; the good, the bad and the ugly.

The A260/230 measurements found IsoG as the only extraction method whose IQR encompassed the optimal value of 2.0, however, this IQR was wide and the median low at 1.545, so although some individual samples may be acceptable, it is overall a poor result. All methods struggled to minimize organic contaminants, and this can greatly impact downstream applications. A260/230 is affected by the salinity of the elution buffer. Increased salt concentration in the DNA sample will lower the A260/230 because of salt absorbance at 230 nm [38]. Both precipitation and silica-binding methods use salts to precipitate and bind DNA, respectively [22]. Additional wash steps may be required to remove excess and contaminating salts.

Traditionally, DNA purity has been prioritised over concentration because of the effects of contamination on downstream applications [39, 40], and prioritising concentration over purity may result in an inaccurate concentration reading due to contaminants. Additional, gentle, washing steps may increase the purity of the sample, if it is required, but there is a risk of decreasing DNA concentration. For samples which contain additional DNA from non-target organisms, concentration may become more of a priority. RL Smith et al. [19] found that because their target ancient powdery mildew DNA was a tiny proportion of the total DNA extracted from the plant specimen, a higher total DNA concentration increased the chance of sequencing powdery mildew DNA. In this study, extracting DNA from individual nematode specimens used the entire worm, additional material cannot be added to improve the DNA concentration and there is likely to be bacteria present due to *T. circumcincta* being collected from the host gut. The methods most successful at PCR amplification were silica binding, and we found these methods had overall higher quality than precipitation or chelating, as has been seen in other studies [17, 18]. NanoDrop 2000™ likely overestimated the concentration of DNA in the samples and the Qubit™ concentration of *T. circumcincta* DNA extracted was low irrespective of the extraction method chosen. As long as purity of a sample is reasonably acceptable, we recommend prioritising a higher DNA concentration over achieving total purity to ensure as much *T. circumcincta* DNA is being extracted as possible because of the presence of host or microorganism DNA that is likely to be present. Calculating correct DNA concentration and purity when concentrations are low is difficult and unlikely to be completely accurate. Additionally, DNA concentration should be based on Qubit™ readings as they are more likely to be a true representation of DNA in a sample than NanoDrop 2000™ readings.

PCR amplification of the ITS-2 gene provides confirmation of correct species DNA extraction, as well as indicating PCR capability of the extraction. For example, impurities such as protein, phenol and other salt traces may terminate a PCR reaction. The methods which had high concentration and purity had the best PCR success, and most methods with low concentration and purity were not PCR successful. There does not appear to be any correlation that indicates a higher concentration or purity of DNA was more successful at PCR amplification. Schi, EznF, AccM and IsoG were all 100% successful at PCR ITS-2 amplification (Fig. 5). Schi, EznF and IsoG PCR success is not unexpected given their generally higher quality DNA. AccM, on the other hand, had low A260/280 (Fig. 3), and very low A260/230 (Fig. 4), but the quality did not seem to affect PCR capability. Interestingly, CTAB had the second lowest concentration on NanoDrop 2000™, was undetectable on Qubit™ and was very low in purity for both A260/280 and A260/230 (Figs. 3, 4), but was still able to produce one successful ITS-2 amplicon for *T. circumcincta* (Tables 2, 3, Fig. 5), indicating that despite the odds, this method can extract *T. circumcincta* DNA, though unreliably. Sequencing of the PCR products had an overall poor outcome; few were sequenced successfully, and the successful fragments were far shorter than expected. Of the four methods of interest, IsoG and Schi shared the greatest median sequence length at 84 bp, much shorter than the target amplicon length of 219 bp. It is possible shearing of the sequences occurred. AccM and Schi extractions produced a sequence of high enough quality only 16.7% of the time. Whereas EznF and IsoG produced sequences of high enough quality 50% of the time. It is unclear why 27 of the PCR products did not meet quality standards, however, it is likely due to the PCR clean-up kit and method used. Initially the PCR products were eluted into TE buffer and stored. When prepared for sequencing, the samples were washed in ethanol and eluted into nuclease-free water. Either the DNA clean-up method, the temporary storage in TE buffer, or the additional wash step has contributed to the background interference and low quality of sequences reported by AGRF.

There are a range of factors here that are influencing sequencing success, and the methods and techniques used in this study have room for optimisation and improvement.

## Conclusions

This study highlights the difficulties in extracting DNA from *T. circumcincta* and that the different extraction methods tested vary significantly in the quality and quantity of DNA recovered. We found the Schi method for extracting DNA from individual *T. circumcincta* nematode specimens to be the best. Schi was able to extract a relatively high concentration of DNA when measured on both NanoDrop 2000™ and Qubit™ (Figs. 1, 2), and measured a tight IQR over the optimal A260/280 (Fig. 3). The CheX method was a strong contender and is a close second, however, it was not as successful at ITS-2 PCR amplification as Schi (Table 3). Considering the purpose is to reliably extract *T. circumcincta* DNA, successful ITS-2 amplification is an important contributing factor. Additionally, the Schi method is simple and fast at approximately 2 h. Exsheathment of the *T. circumcincta* cuticle is unnecessary. Although not tested in this study, it is likely these results could be generalized to related species such as *Haemonchus contortus* or *Ostertagia ostertagi*. Further optimization of methods for extracting *T. circumcincta* DNA is required.

## Methods

### Sampling

*Teladorsagia circumcincta* specimens were harvested from experimentally infected sheep at Federation University, Australia). Animals were bred and supplied on-site by the Gippsland Field Station, owned and operated by Monash University. Animal ethics approval was approved by Federation University (AEC # 17008). 5–6-month-old Merino wethers were infected with 5000 *T. circumcincta* third-stage larvae. Approximately 5 weeks post infection, adult parasites were collected from the abomasum. The animals were euthanized (humanely killed) with a lethal intravenous dose (5 g) of pentobarbitone (Lethobarb®, Virbac Pty Ltd., Sydney, Australia) administered by jugular venepuncture. This is a standard method to induce euthanasia in animals. The abomasum was removed from the animal and opened along the greater curvature to reveal the gastric mucosa. The abomasum was then placed in a 50 × 30 cm plastic tray and gently rinsed with saline to remove the contents. All folds of the abomasum were carefully examined to collect all parasites. Parasites dislodged from the abomasal surface following washing were collected with forceps. All collected parasites were washed in PBS 3 times at 4 °C. *T. circumcincta* specimens were 4separated by sex by light microscopy and stored in 100% ethanol at 4 °C and washed in MilliQ H_2_O immediately prior to use.

### DNA extraction

Ten DNA extraction protocols were selected for comparison, which encompassed the most common DNA extraction methods, including chelating, silica binding and precipitation (Table 1). An exsheathment step prior to DNA extraction was applied to 1 DNA extraction protocol to determine whether cuticle removal improved DNA yield, bringing the total protocols compared to 11. The methods were performed as per both manufacturer’s instructions and previously published DNA extraction protocols. Six individual adult, female nematode specimens were used for each extraction method. Males were excluded for a separate study. Detailed methods are outlined in Additional File 2. All methods were eluted to 50 μL. DNA was quantified using two methods, Qubit™ fluorometer (Life Technologies, Singapore) and NanoDrop 2000™ spectrophotometer (Thermo Fisher Scientific, Wilmington, Delaware, USA). The NanoDrop 2000™ was blanked using the respective elution buffer for the method. All DNA extraction product concentrations were measured using Qubit™ (1X dsDNA HS (High Sensitivity) Assay Kit, Invitrogen, #Q33231) and NanoDrop 2000™, and purity was measured using the 260/280 nm and 260/230 nm absorbency ratios of NanoDrop 2000™.

### Statistical analysis

Statistical analyses were performed using R version 4.03 [41] and package FSA [42]. The distribution of the data was determined to be non-normally distributed using the graphics package and function ‘hist’. The function ‘kruskal.test’ was used to perform a Kruskal-Wallis non-parametric test to identify significant differences among DNA extraction methods in DNA concentration when measured on NanoDrop 2000™ spectrophotometer and Qubit™ fluorometer, 260/280 nm absorbance ratio, and 260/230 nm absorbance ratio. The function ‘dunn.test’ was used to perform a post-hoc Dunn’s Multiple Comparison Test to identify significant pairwise comparisons. Variables were considered significant with *P* ≤ 0.05. *P*-values adjusted with the Holm method.

### PCR amplification and sequencing of the rRNA ITS-2 from individual nematodes

An approximately 219 bp expected fragment size encompassing partial ITS-2 and 28S ribosomal RNA sequences was amplified by PCR from individual nematodes using the ITS-2 (5′-TATGCAACATGACGTACGACGG-3′) and NC2 (5′-TTAGTTTCTTTTCCTCCGC-3′) primers described in NJ Bott et al. [31]. PCR reaction conditions were 12.5 μL GoTaq Green Master Mix (Promega, M7122), 0.5 μL ITS-2 primer (10 μM), 0.5 μL NC2 primer (10 μM), 2.5 μL DNA template, and 9 μL nuclease-free H_2_O. The negative control contained nuclease-free H_2_O, and the positive control contained the following: a gBlocks™ Gene Fragment (Integrated DNA Technologies, Coralville, USA) of the last 219 bp of *Teladorsagia circumcincta* 18S ribosomal RNA gene, partial sequence; internal transcribed spacer 1, 5.8S ribosomal RNA gene, and internal transcribed spacer 2, complete sequence; and 28S ribosomal RNA gene, partial sequence (GenBank accession: JF680984) in place of the DNA template, respectively. The thermocycling parameters were 95 °C for 2 min, followed by 50 cycles of 95 °C for 15 s, 60 °C for 30 s, 72 °C for 15 s followed by a final extension of 72 °C for 5 min. PCR products were confirmed on 1% agarose gel. Positive PCR products were purified with a Wizard® SV Gel and PCR Clean-Up System (Promega, A9281) and temporarily stored in TE buffer at 4 °C. PCR products were washed in ethanol and eluted into nuclease-free water prior to being sent to the Australian Genome Research Facility and directly sequenced using Sanger sequencing with the ITS-2 primer. Sequences were aligned using Geneious version 2020.2 created by Biomatters. Available from https://www.geneious.com.

Sequenced PCR products which passed QC (≥Q20) were aligned with the PCR positive control using Geneious Prime software.

## Supplementary Information

**Additional file 1: Table S1.** Statistically significant differences in NanoDrop 2000™ and Qubit™ DNA concentration, and 260/230 nm absorbance ratio using the 11 DNA extraction methods according to the Dunn’s Multiple Comparison Test.

**Additional file 2.** DNA extraction methods.

## Data Availability

All data generated or analyzed during this study are included in this article and its supplementary information files (Additional files 1, 2) or are available in the GenBank repository (GenBank accessions MW161470-MW161483). Raw datasets used and/or analyzed during the current study are available from the corresponding author.

## Abbreviations

DNA: Deoxyribonucleic acid
PCR: Polymerase chain reaction
AccM: AccuPrep® Genomic DNA Extraction - Mammalian Tissue
AccW: AccuPrep® Genomic DNA Extraction Kit – Whole Blood, Buffy Coat and Cultured Cells
CheX: Chelex®100
CTAB: Cetyl trimethyl ammonium bromide
EznF: E.Z.N.A.® Forensic DNA
IsoG: Isolate II Genomic DNA Kit
Schi: *Schistosoma sp.* DNA Extraction Method
Schi-LE: Schi with larval exsheathment
SDS: sodium dodecyl sulphate
WizM: Wizard® Genomic DNA Purification Kit - Mouse Tail
WizP: Wizard® Genomic DNA Purification - Plant Tissue
ITS-2: Internal transcribed spacer region 2
A260/280: 260 nm / 280 nm absorbency ratio
A260/230: 260 nm / 230 nm absorbency ratio
Q20: Q-score 20
QC: Quality check
T: Thymine
C: Cytosine
G: Guanine
TE buffer: Tris-EDTA buffer
AGRF: Australian Genome Research Facility
PBS: Phosphate-buffered saline
rRNA: Ribosomal ribonucleic acid
