# Supplementary material for: Comparative evaluation of different molecular methods for DNA extraction from individual Teladorsagia circumcincta nematodes
Source: BMC Biotechnol. 2021 May 17;21:35. doi: 10.1186/s12896-021-00695-6 (PMC8130168; doi:10.1186/s12896-021-00695-6)
Supplement: Supplementary file 1 — Additional file 1: Table S1. Statistically significant differences in NanoDrop 2000™ and Qubit™ DNA concentration, and 260/230 nm absorbance ratio using the 11 DNA extraction methods according to the Dunn’s Multiple Comparison Test. [file 12896_2021_695_MOESM1_ESM.docx]

Table AF1: Statistically significant differences in NanoDrop 2000™ and Qubit™ DNA concentration, and 260/230 nm absorbance ratio using the 11 DNA extraction methods according to the Dunn’s Multiple Comparison Test.

| Method Comparison | |  | |  | |
| --- | --- | --- | --- | --- | --- |
| NanoDrop 2000™ DNA Concentration | | Z | P. unadj | | P. adj |
| AccM | CheX | -4.03309 | 5.50E-05 | | 2.86E-03 |
| CheX | CTAB | 4.665147 | 3.08E-06 | | 1.67E-04 |
| CTAB | EznF | -3.64935 | 2.63E-04 | | 1.26E-02 |
| CTAB | Schi | -3.83746 | 1.24E-04 | | 6.22E-03 |
| CheX | Schi-LE | 3.574104 | 3.51E-04 | | 1.65E-02 |
| CTAB | SDS | -3.49886 | 4.67E-04 | | 2.15E-02 |
| CheX | WizM | 4.9586 | 7.10E-07 | | 3.91E-05 |
| EznF | WizM | 3.942802 | 8.05E-05 | | 4.11E-03 |
| Schi | WizM | 4.130912 | 3.61E-05 | | 1.92E-03 |
| SDS | WizM | 3.792313 | 1.49E-04 | | 7.31E-03 |
| Qubit™ DNA Concentration | |  |  | |  |
| AccM | CheX | -3.56893 | 3.58E-04 | | 0.012187 |
| AccW | CheX | -3.92687 | 8.61E-05 | | 0.003098 |
| AccM | Schi | -3.50576 | 4.55E-04 | | 0.015025 |
| AccW | Schi | -3.86371 | 1.12E-04 | | 0.003909 |
| CheX | Schi-LE | 3.386779 | 7.07E-04 | | 0.02263 |
| Schi | Schi-LE | 3.326552 | 8.79E-04 | | 0.027258 |
| A260/230 | |  |  | |  |
| Optimal* | Schi-LE | 3.898243 | 9.69E-05 | | 0.006007 |
| EznF | SDS | 4.029334 | 5.59E-05 | | 0.00358 |
| IsoG | SDS | 4.029334 | 5.59E-05 | | 0.003524 |
| Optimal* | SDS | 4.70549 | 2.53E-06 | | 0.000167 |
| EznF | WizM | 3.725754 | 1.95E-04 | | 0.011879 |
| IsoG | WizM | 3.725754 | 1.95E-04 | | 0.011684 |
| Optimal* | WizM | 4.40191 | 1.07E-05 | | 0.000697 |

* Optimal ratio for pure DNA using A260/230 measurement is 2.0.
Z: Values for the Z test statistic for each comparison.
P. unadj: Unadjusted p-values for each comparison.
P. adj: Adjusted p-values for each comparison.
