# Supplementary material for: Comparative evaluation of different molecular methods for DNA extraction from individual Teladorsagia circumcincta nematodes
Source: BMC Biotechnol. 2021 May 17;21:35. doi: 10.1186/s12896-021-00695-6 (PMC8130168; doi:10.1186/s12896-021-00695-6)
Supplement: Supplementary file 2 — Additional file 2. DNA extraction methods. [file 12896_2021_695_MOESM2_ESM.docx]

Additional File 2

# DNA extraction methods

## AccM

AccuPrep Genomic DNA Extraction – Mammalian Tissue

Disrupt or homogenise the sample into a clean 1.5 ml tube and add 200 μL of Tissue Lysis buffer. Add 20 μL of Proteinase K, mix by vortex mixer, and incubate at 60°C for 1 hour or until the tissue is completely lysed. Briefly spin down to remove drops from inside the lid and sides. Add 200 μL Binding buffer and immediately vortex. Incubate at 60°C for 10 mins. Add 100 μL isopropanol and mix well by pipetting. Carefully transfer the lysate into the binding column tube. Centrifuge at 8,000 rpm for 1 min. Transfer the binding column tube to a new 2 ml tube. Add 500 μL Washing buffer 1 and centrifuge at 8,000 rpm for 1 min. Dispose of flow-through. Add 500 μL of Washing buffer 2 and centrifuge at 8,000 rpm for 2 mins. Transfer the binding column tube to a clean 1.5 ml tube. Add 30 μL of Elution buffer and incubate at room temperature for 1 min. Centrifuge at 8,000 rpm for 1 min.

## AccW

AccuPrep Genomic DNA Extraction – Whole Blood, Buffy Coat and Cultured Cells

Add 20 μL of Proteinase K to a clean 1.5 ml tube. Add sample, 200 μL of Binding buffer and vortex immediately. Incubate at 60°C for 10 mins. Add 100 μL isopropanol and mix well by pipetting. Carefully transfer the lysate into the binding column tube. Centrifuge at 8,000 rpm for 1 min. Transfer the binding column tube to a new 2 ml tube. Add 500 μL Washing buffer 1 and centrifuge at 8,000 rpm for 1 min. Dispose of flow-through. Add 500 μL of Washing buffer 2 and centrifuge at 8,000 rpm for 2 mins. Transfer the binding column tube to a clean 1.5 ml tube. Add 30 μL of Elution buffer and incubate at room temperature for 1 min. Centrifuge at 8,000 rpm for 1 min.

## CheX

Chelex-100

Aliquot 300 μL 5% Chelex solution into 1.5 ml tubes. Under dissection microscope, place sample on a slide and crush with another clean microscope slide. Use a needle-stick to collect crushed sample and place into tube with 5% Chelex. Incubate tubes at 56°C for 15 mins, vortex thoroughly then incubate at 100 °C for 8 mins. Vortex tubes and centrifuge at 15,000 rpm for 5-10 mins. Transfer 200 μL of supernatant into new 1.5 ml tube.

## CTAB

CTAB DNA Extraction

Add 500 μL 2X CTAB buffer (100 mM Tris-HCl pH 8, 1.4 M NaCl, 20 mM EDTA, 2% CTAB, 0.02 g / 1 ml PVP-40) to 2 ml tube with sample. Using pellet pestle, grind sample. Perform short grinds (30 seconds max.) followed by cooling on ice to avoid heat damage to DNA. Add 500 μL 2X CTAB buffer heated to 65°C and incubate at 65°C for 60 mins. Add 750 μL SEVAG (24:1 chloroform:isoamyl alcohol) and mix gently on orbital shaker for up to 1 hour. Centrifuge at 13,000 rpm for 10 mins. Transfer top aqueous phase to a new 1.5 ml tube. Add 750 μL SEVAG and mix gently on orbital shaker for up to 1 hour. Centrifuge at 13,000 rpm for 10 mins. Transfer top aqueous phase to a new 1.5 ml tube. Add 2/3 volume of -20°C isopropanol and mix gently. Centrifuge at 13,000 rpm for 10 mins. Pour off liquid in waste container in fume hood. Add 750 μL of ice-cold 70% ethanol, shake well, and wash for 60 mins. Centrifuge at 13,000 rpm for 5 mins. Pour off liquid and drain upside-down for 5-10 mins. If ethanol still present in tubes do an extra spin in centrifuge and pipette ethanol out carefully. Resuspend DNA in 100 μL TE buffer pH 8 (10 mM Tris-HCl, 1 mM EDTA). Add 1 μL 100 mg/ml RnaseA and vortex. Incubate at 37°C for 20 mins. Add 100 μL of cold 7.5 M ammonium acetate and 750 μL 100% ethanol. Incubate on ice for 30 mins. Centrifuge ar 13,000 rpm for 10 mins. Pour off liquid in waste container in fume hood. Add 500 μL ice-cold 70% ethanol and shake well. Wash for 30 mins. Centrifuge at 13,000 rpm for 5 mins. Pour off supernatant and air-dry upside-down for 5-10 mins. Dissolve DNA in 50 μL dH_2_O.

## EznF

E.Z.N.A.® Forensic DNA Kit – Standard Protocol

Place sample into 1.5 ml tube, add 200 μL TL Buffer and vortex. Incubate at 55°C for 15 mins. Vortex every 2 mins. Add 25 μL OB Protease Solution and vortex. Incubate at 60°C for 45 mins with occasional mixing. Centrifuge at maximum speed to collect any sample adhering to walls. Add 225 μL BL Buffer and vortex. Incubate at 60°C for 10 mins. Centrifuge at max. speed to collect any sample adhering to walls. Add 300 μL 100% isopropanol and vortex. Centrifuge at max. speed to collect any sample adhering to walls. Insert column to 2 ml collection tube. Add 100 μL 3 M NaOH to column and incubate at room temperature for 4 mins. Centrifuge at max. speed for 30 secs. Discard filtrate and reuse collection tube. Transfer sample to column and centrifuge at max. speed for 1 min. Discard filtrate and collection tube. Transfer column to new 2 ml collection tube, add 500 μL HBC Buffer and centrifuge at max. speed for 1 min. Discard filtrate and collection tube. Transfer column to a new 2 ml collection tube, add 700 μL DNA Wash Buffer and centrifuge at max. speed for 1 min. Discard the filtrate and reuse the collection tube. Do a second wash by adding 700 μL DNA Wash Buffer and centrifuge at max. speed for 2 mins. Place the column into a clean 1.5 ml tube and add 30 μL Elution Buffer heated to 70°C. Incubate at room temperature for 3 minutes and then centrifuge at max. speed for 1 min.

## IsoG

ISOLATE II Genomic DNA Kit

Add 180 μL Lysis Buffer GL and sample to 1.5 ml tube. Add 25 μL Proteinase K and vortex. Incubate at 56°C for 1-3 hours, until completely lysed, vortex occasionally. Add 200 μL Lysis Buffer G3 and vortex vigorously. Incubate at 70°C for 10 mins. Vortex briefly and add 210 μL 100% ethanol, vortex again. Place spin column in 2 ml collection tube and load sample to the column. Centrifuge at 11,000 x *g* for 1 min. Discard flow-through and reuse collection tube. Add 500 μL Wash Buffer GW1 to column and centrifuge for 1 min at 11,000 x *g*. Discard the flow-through and reuse the collection tube. Add 600 μL Wash Buffer GW2 and centrifuge for 1 min at 11,000 x *g*. Discard the flow-through and reuse collection tube. Centrifuge for 1 min at 11,000 x *g* to remove residual ethanol. Place column in a clean 1.5 ml tube. Add 30 μL Elution Buffer G preheated to 70°C to column. Incubate at room temperature for 1 min. Centrifuge for 1 min at 11,000 x *g*.

## Schi

*Schistosoma sp.* DNA Extraction

Mix Homogenisation Buffer (100 mM NaCl, 200 mM sucrose, 10 mM EDTA, 30 mM Tris pH 8) and Lysis Buffer (250 mM EDTA pH 8, 2.5% SDS, 500 mM Tris pH 9.2) at 4:1 to create fresh Grinding Buffer (GB). Grind 1 nematode in 25 μL GB in 1.5 ml tube. Rinse the pestle with 25 μL of fresh GB and add wash to sample. Incubate at 65°C for 30 mins. Add 8 M ammonium acetate to a final concentration of 1 M. Incubate on wet ice for 30-60 mins. Centrifuge at 13,000 rpm for 10 mins. Transfer supernatant to a clean 1.5 ml tube. Add 100 μL 100% ethanol, mix, and incubate at room temperature for 5 mins. Centrifuge for 15 mins at 13,000 rpm at 4°C. Remove ethanol carefully. Wash barely visible pellet with 100% ethanol once, remove ethanol carefully and let tube air-dry but not over dry. Add 30 μL TE buffer (10 mM Tris, 1 mM EDTA, pH 8).

## SDS

Sodium Dodecyl Sulphate (SDS) DNA Extraction

Add 150 μL of DNA extraction buffer (200 mM Tris pH 8, 250 mM NaCl, 125 mM EDTA, 0.5% SDS) and sample to 1.5 ml tube, and crush with a sterile pellet pestle. Add 150 μL of DNA extraction buffer to was pellet pestle into the tube. Vortex for 30 sec. Freeze at -20°C for 10 mins. Incubate at 70°C for 10 mins, vortex at 5 mins. Incubate at 4°C for 5 mins. Centrifuge at 13,000 rpm for 10 mins at 4°C. Transfer the supernatant to a new 1.5 ml tube containing 300 μL ice-cold isopropanol. Mix by pipetting. Centrifuge at 13,000 rpm for 10 mins at 4°C. Remove and discard the supernatant without disturbing the pellet. Wash the DNA pellet with 300 μL of ice-cold ethanol and mix by pipetting. Centrifuge at 13,000 rpm for 10 mins at 4°C. Remove and discard ethanol supernatant without disturbing the pellet and air-dry tube until all traces of ethanol have evaporated. Resuspend DNA pellet in 30 μL dH­­_2_O.

## WizM

Wizard Genomic DNA Purification Kit – Animal Tissue (Mouse Tail)

Add 120 μL of 0.5 M EDTA (pH 8) and 500 μL of Nuclei Lysis Solution to a 1.5 ml tube and chill on ice. Add sample to a new 1.5 ml tube and add 600 μL of EDTA/Nuclei Lysis Solution. Add 17.5 μL of 20 mg/ml Proteinase K. Incubate at 55°C for 3 hours, vortex the sample once per hour. Add 3 μL of RNase Solution and mix by inversion 2-5 times. Incubate at 37°C for 30 mins. Cool to room temperature for 5 mins. Centrifuge for 4 mins at 13,000 x *g*. Transfer the supernatant to a clean 1.5 ml tube containing 600 μL isopropanol. Mix by inversion. Centrifuge for 1 min at 13,000 x *g*. Remove and discard of the supernatant carefully. Air-dry the pellet for 10-15 mins. Add 30 μL Rehydration Solution and incubate at 4°C overnight.

## WizP

Wizard Genomic DNA Purification Kit – Plant Tissue

Add sample to 600 μL of Nuclei Lysis Solution and vortex for 1-3 secs. Incubate at 65°C for 15 mins. Add 3 μL RNase Solution and mix by inversion. Incubate at 37°C for 15 mins. Cool to room temperature for 5 mins. Add 200 μL of Protein Precipitation Solution and vortex vigorously for 20 seconds. Centrifuge for 3 mins at 13,000 x *g*. Transfer supernatant to a clean 1.5 ml tube containing 600 μL of isopropanol. Mix by inversion. Centrifuge for 1 min at 13,000 x *g*. Remove and dispose of supernatant. Add 600 μL of 70% ethanol and invert gently. Centrifuge at 13,000 x *g* for 1 min. Remove and discard of the supernatant carefully. Air-dry the pellet for 10-15 mins. Add 30 μL Rehydration Solution and incubate at 4°C overnight.

## -LE

Larval exsheathment

Nematodes washed twice by centrifugation in distilled water at 300 x *g* for 5 mins. Discard supernatant between washes. Pellet sample by centrifugation at 13,000 x *g* for 30 secs. Resuspend in 400 μL distilled water. Add 400 μL 1% sodium hypochlorite, to give a final conc. of 1.5%. Incubate at 40°C for 10 mins. Remove supernatant and wash in distilled water. Centrifuge at 13,000 x *g* for 30 secs. Discard supernatant and wash again in distilled water. Centrifuged at 13,000 x *g* for 30 secs. Discard supernatant and resuspend in 400 μL distilled water. Incubate at 40°C in a 10% CO_2_ atmosphere for 30 mins. Store at 4°C until required for DNA extraction.
